# First Isolation of *Candida parapsilosis* from Water-Related Domestic Environments in Colombia: A Public Health Concern?

**DOI:** 10.3390/jof12090694

**Published:** 2026-09-16

**Authors:** Norida Vélez, Valentina Valencia, Yohana M. Velasco-Santamaría, Patricia Escandón, María José Jiménez-A, Andrés Ceballos-Garzon

**Affiliations:** 1 Grupo de Investigación en Biotecnología y Toxicología Acuática y Ambiental—BioTox, Escuela de Ciencias Animales, Facultad de Ciencias Agropecuarias y Recursos Naturales, Universidad de los Llanos, Villavicencio 500017, Colombia; valentina.valencia@unillanos.edu.co (V.V.); ymvelascos@unillanos.edu.co (Y.M.V.-S.); 2 Grupo de Microbiología, Instituto Nacional de Salud, Bogotá 111321, Colombia; escandonp@gmail.com; 3 Grupo de Investigación Ceparium, Facultad de Ciencias de la Salud, Universidad Colegio Mayor de Cundinamarca, Bogotá 110311, Colombia; mariajjimenez@universidadmayor.edu.co; 4 Studies in Translational Microbiology and Emerging Diseases (MICROS) Research Group, School of Medicine and Health Sciences, Universidad del Rosario, Bogotá 111221, Colombia; carlos.ceballos@urosario.edu.co

**Keywords:** *Candida parapsilosis*, domestic water, fluconazole resistance, *ERG*11, One Health

## Abstract

Background: *Candida parapsilosis* is an opportunistic yeast of increasing clinical importance, with fluconazole resistance rates rising globally and reaching concerning levels in clinical settings across several countries, including Colombia. However, the occurrence of antifungal-resistant *Candida* species in domestic water systems in the country remains largely unexplored. Methods: This cross-sectional study assessed the occurrence and antifungal susceptibility of *Candida* spp. in intradomiciliary water from 104 households in Villavicencio, Colombia, during two monitoring periods. Water samples were filtered, and yeast-like colonies were identified using MALDI-TOF mass spectrometry. Susceptibility to fluconazole, amphotericin B, and anidulafungin was determined using the CLSI broth microdilution method. The *ERG*11 gene was sequenced in the fluconazole-resistant isolate. Results: *C. parapsilosis* was recovered from 16 of 104 households (15.4%), yielding 22 isolates distributed across six communes. Most isolates (72.7%) were obtained from household water storage tanks. One isolate was resistant to fluconazole (MIC = 8 µg/mL) and harbored a nonsynonymous R398I substitution in *ERG*11. Conclusions: To our knowledge, this is the first report of *C. parapsilosis*, including a fluconazole-resistant isolate carrying an *ERG*11 substitution, recovered from domestic water in Colombia. These findings suggest that household water storage tanks may serve as environmental reservoirs for this opportunistic yeast. The implications for human exposure remain to be determined and highlight the need for integrated One Health surveillance.

## 1. Introduction

Yeasts constitute an important component of the microbial community in aquatic environments [1]. Several pathogenic or opportunistic yeasts have been isolated from diverse aquatic matrices, including lakes [2], rivers [3,4], drinking water [5,6], and wastewater [7,8,9]. Importantly, these environmental yeasts are susceptible to developing resistance to antifungal agents, including fluconazole, which remains a first-line therapeutic agent in many low- and middle-income countries where access to echinocandins is limited [10,11].

In recent years, reports of antifungal-resistant yeast strains commonly found in aquatic environments have become increasingly frequent. For instance, Medeiros et al. [12] isolated *Candida* spp. resistant to itraconazole and fluconazole from lakes and rivers in southeastern Brazil. Similarly, Milanezi et al. [8] conducted a study in Rio Grande do Sul, Brazil, and reported a high prevalence of *Candida* spp. and *Debaryomyces* spp. resistant to azole antifungals and amphotericin B. Across South America, studies have documented antifungal-resistant isolates of pathogenic yeasts from lakes, lagoons, rivers, and wastewater treatment plants [1].

Among the species within the genus *Candida* spp., *C. parapsilosis*, alongside the emerging multidrug-resistant pathogen *C. auris*, has garnered particular concern owing to its capacity to colonize aquatic environments and its well-documented role in healthcare-associated infections [13]. *C. parapsilosis* is responsible for invasive infections predominantly in immunocompromised patients [14] and, of particular concern, fluconazole resistance is escalating globally. In Colombia, recent data have reported high resistance rates among clinical isolates [15,16]. Although its clinical prevalence has been widely documented, a significant knowledge gap persists regarding its distribution in aquatic environmental matrices in Latin America [17]. Globally, the presence of *C. parapsilosis* in water is frequently reported [18], where it acts as a colonizer of household surfaces composed of plastic, rubber, and silicone matrices. This environmental persistence establishes a potential reservoir for onward transmission to vulnerable populations.

In Colombia, routine microbiological surveillance for *Candida* spp. in intradomiciliary water remains limited. Although some studies have documented yeast diversity in aquatic matrices [19,20], no previous study has specifically reported the presence of *C. parapsilosis* in water intended for domestic use in Colombian cities. The quality of drinking water and its association with microbiological contamination represent an important public health concern [21].

This local knowledge gap mirrors a broader regional pattern: socio-environmental pressures, including substandard housing quality and inequitable access to safe water, have been proposed as drivers of *Candida* spp. transmission outside hospital settings across Latin America [17]. Conversely, in other regions, *C. parapsilosis* ranks among the yeasts most frequently recovered from groundwater intended for human consumption [22].

Therefore, the present study aimed to describe the occurrence of *Candida* spp. in intradomiciliary water samples collected from households across Villavicencio, an urban area of approximately 600,000 inhabitants located in central-eastern Colombia.

## 2. Materials and Methods

This study was conducted within the framework of a macro-project, which sought to identify pathogenic microorganisms of public health interest in intradomiciliary water across the city of Villavicencio.

### 2.1. Study Population, Design, Sample Size Calculation, and Eligibility Criteria

A cross-sectional study was conducted in the city of Villavicencio, Department of Meta, Colombia. Two monitoring campaigns were carried out, corresponding to distinct climatic periods: the period of increasing rainfall (August 2025) and the transition to the dry season (late November 2025). The study population comprised urban households with access to an intradomiciliary water distribution system across the eight communes of the city.

For household selection, the community was invited to participate through digital recruitment campaigns. For the registered households, a stratified sampling approach was employed, selecting 13 households from each commune. This yielded a final sample size of 104 households.

Households were included if they possessed an active intradomiciliary water supply system and were located within any of the eight communes of Villavicencio. Institutional facilities, including hospitals, clinical laboratories, schools, universities, and daycare centres, were excluded from the study.

### 2.2. Sample Collection

For each household visited, subject to water availability at the time of the visit, two water samples were collected: one from a faucet supplied directly by a public or community water supply system and the other from a faucet connected to a water storage tank. Before sample collection, the external surface of the faucet was disinfected with alcohol. When feasible, the inner surface was also cleaned using a swab to facilitate access. The faucet was then allowed to run for approximately 45 s to flush stagnant water from the plumbing and ensure that the sample was representative of the corresponding water source. After flushing, the pH was measured on site using a pH indicator strip before the sample was collected. During sample collection, a flame was maintained near the faucet to minimise airborne contamination.

Approximately 200–300 mL of water was collected in Nasco sampling bags containing sodium thiosulfate tablets, which were used to neutralise residual chlorine and prevent interference with bacterial recovery. At least one inch of headspace was left between the water surface and the bag closure to maintain aeration and allow adequate mixing of the sample. The samples were subsequently refrigerated at 2–8 °C and transported in expanded polystyrene coolers with ice packs to protect them from spills, physical damage, light exposure, and heat. Each sample was labelled in the white write-on area of the Nasco bag, indicating the district, sampling number, collection point, and sample source. Samples were processed within approximately 18 h of collection.

A total of 348 water samples were collected from 104 households across the two monitoring periods. During the first period (transitional rainfall), 208 samples were obtained: 156 (75.0%) from water storage tanks and 52 (25.0%) from taps directly connected to the public or community supply. During the second period (ascending rainfall), 140 samples were collected: 97 (69.3%) from storage tanks and 43 (30.7%) from direct supply. Overall, 253 samples (72.7%) were from storage tanks and 95 samples (27.3%) from direct connections. Not all households yielded both sample types because water availability from the public network varied at the time of the visit.

### 2.3. Sample Processing: Membrane Filtration

It should be noted that this investigation was nested within a broader water quality monitoring program primarily designed for the recovery of bacterial pathogens (findings to be reported separately). Consequently, the filtration and culture protocol was standardised for the detection of enteric bacteria. Briefly, a volume of 200 mL of water was filtered under vacuum through a cellulose nitrate membrane with a pore size of 0.45 µm (Millipore^®^, Burlington, MA, USA). The membrane was then transferred aseptically to a Petri dish containing MacConkey agar or nutrient agar. Both media were incubated at 37 °C for 24–48 h. Yeast-like colonies were recovered as incidental isolates (nutrient agar) and subsequently preserved in skim milk at −20 °C for downstream identification by Matrix-Assisted Laser Desorption/Ionization Time-Of-Flight Mass Spectrometry (MALDI-TOF MS; MALDI Biotyper, Bruker Daltonics, Bremen, Germany).

### 2.4. Matrix-Assisted Laser Desorption/Ionization Time-of-Flight Mass Spectrometry (MALDI-TOF MS)

Preserved isolates were streaked from glycerol stocks onto Sabouraud Dextrose Agar (SDA) and grown for 24–36 h at 35 °C. Protein extraction was performed using the formic acid/ethanol method, according to the Bruker Daltonics’ protocol. Mass spectra were acquired and analyzed using the Flex Control software version 3.4 and the MALDI Biotyper version 3.1 (Bruker Daltonics, Bremen, Germany). Identification scores were interpreted according to the manufacturer’s specifications: correct genus and species identification (score ≥ 2.0), reliable genus identification (score 1.7–2.0), and no reliable identification (score < 1.7). All isolates processed in this study yielded scores above 2.0 [23].

### 2.5. Antifungal Susceptibility Testing

Susceptibility to amphotericin B (AMB), fluconazole (FLC), and anidulafungin (AND) (Sigma-Aldrich, St. Louis, MO, USA) was determined using the Clinical and Laboratory Standards Institute broth microdilution (CLSI-BMD) method, following the M27 4th edition guidelines and interpreted according to the M27M44S document [24,25]. Quality control was ensured by testing the CLSI-recommended reference strains *C. parapsilosis* ATCC 22019 and *C. krusei* ATCC 6258.

Interpretive criteria were applied according to CLSI guidelines [24]. Resistance to fluconazole and anidulafungin was defined as a Minimum Inhibitory Concentration (MIC) ≥ 8 µg/mL. For amphotericin B, the Epidemiological Cutoff Value (ECV) of 1 µg/mL was applied. MIC endpoints for fluconazole and anidulafungin were defined as the lowest drug concentration that produced a prominent reduction in visual growth (approximately 50% inhibition) relative to the drug-free growth control. For amphotericin B, the MIC was defined as the lowest concentration that resulted in complete inhibition of visual growth (100% inhibition).

### 2.6. Sequencing Analysis of ERG11

The fluconazole-resistant isolate was further characterized by amplification and bidirectional sequencing of the entire *ERG*11 gene coding region. PCR was performed using a single-tube protocol described previously [26]. Amplicons were purified and sequenced on a SeqStudio genetic analyser (Applied Biosystems, Foster City, CA, USA). The resulting sequences were analysed using BLAST (https://www.MycoBank.org/) and aligned against the *C. parapsilosis* reference sequence for *ERG*11 available in GenBank (accession no. GQ302972) to identify known resistance-associated amino acid substitutions.

### 2.7. Statistical Analysis

A descriptive analysis of the data was performed using Stata version 19 (StataCorp LLC, College Station, TX, USA). Fisher’s exact test was used to compare the frequency of *C. parapsilosis* isolation between the two monitoring periods. Statistical significance was defined as a two-sided *p*-value < 0.05.

### 2.8. Ethical and Legal Considerations

The study was approved by the Institutional Research Ethics Committee of Universidad de los Llanos (Minutes No. 04, dated 5 April 2024). Written informed consent was obtained from the heads of all participating households before sample collection. Participant confidentiality was ensured by assigning a unique numerical identification code to each household. Geographic location, water source type, and basic household demographic data were recorded using a standardised form and subsequently anonymised.

## 3. Results

### 3.1. Recovery and Distribution of Candida parapsilosis

A total of 348 water samples collected from participating households during the two follow-up campaigns were analyzed: 208 were collected during the ascending rainfall period and 140 during the transition period. According to the water supply source, 95 samples were collected from faucets directly connected to public or community water supply systems (52 during the ascending rainfall period and 43 during the transition period). The remaining 253 samples were collected from faucets connected to water storage tanks (156 during the ascending rainfall period and 97 during the transition period). Water availability from public or community supply systems varied among participating households, limiting the collection of samples directly from the distribution network. Consequently, most samples were obtained from household storage systems, which typically consisted of an underground reservoir from which water was pumped to an elevated tank and subsequently distributed throughout the household by gravity.

**Figure 1:**
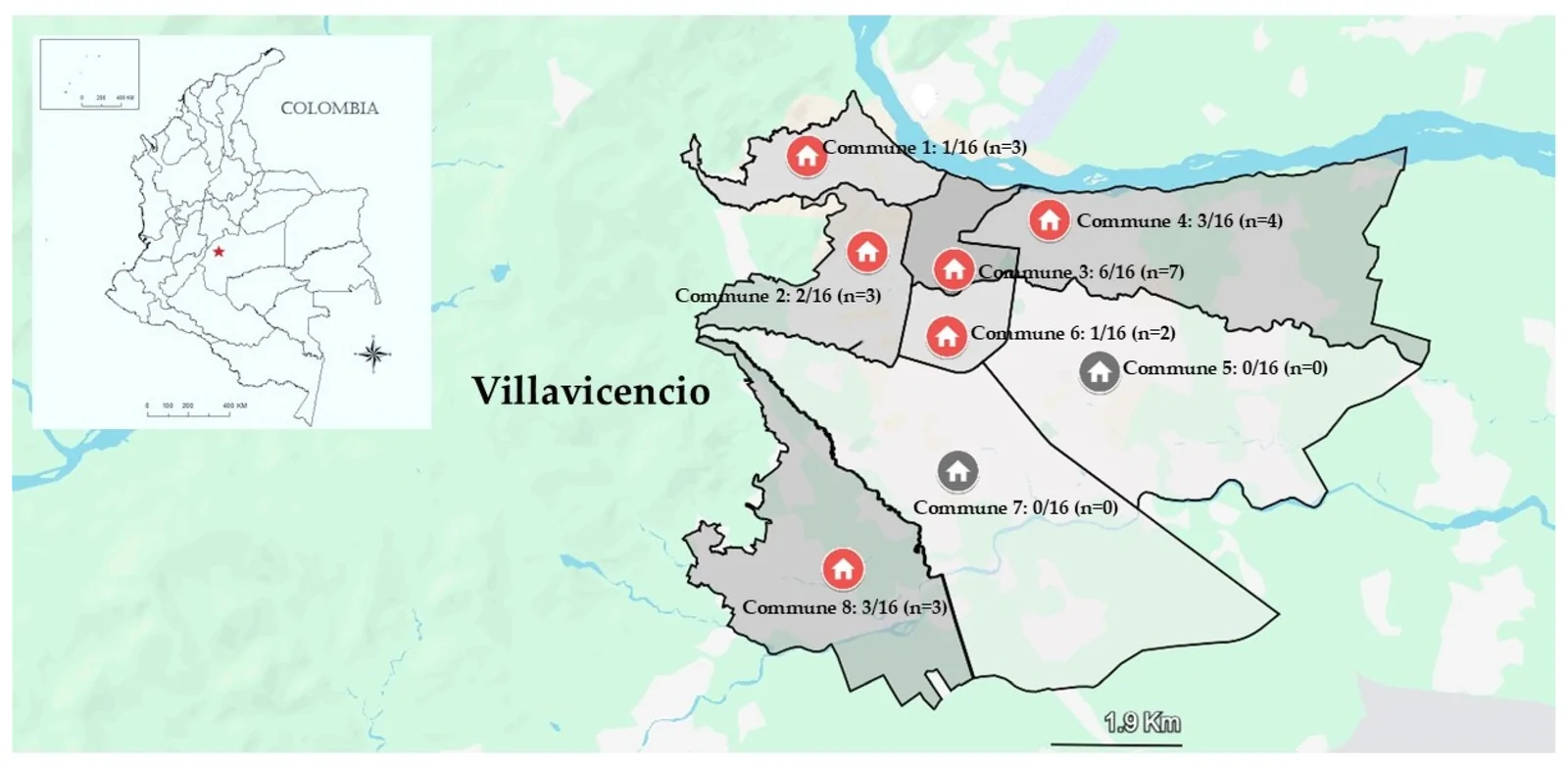
Geographic distribution of *Candida parapsilosis*-positive households across the eight communes of Villavicencio, Colombia. Red house icons indicate communes in which at least one *C. parapsilosis* isolate was recovered from intradomiciliary water (Communes 1, 2, 3, 4, 6, and 8); grey house icons indicate communes with no isolates recovered (Communes 5 and 7). The red star represents the location of Villavicencio within Colombia. Numbers within each commune are expressed as the number of positive households relative to the total number of positive households identified across all communes (n = 16), followed in parentheses by the number of isolates recovered in that commune. Note that the isolate count may exceed the positive-household count, as some households yielded more than one isolate. Scale bar = 1.9 km.

Among the species of the genus *Candida*, only *C. parapsilosis* (n = 22) was recovered, identified in 16 out of the 104 (15.4%) participating households. The 22 isolates were recovered across both monitoring periods, with 11 isolates obtained in each period. Isolates were obtained from six of the eight communes of Villavicencio (C1, C2, C3, C4, C6, and C8) (Figure 1). No statistically significant differences were observed in isolation frequency between the two periods (Fisher’s exact test, *p* = 0.372).

Commune 3 accounted for the largest proportion of isolates (31.8%; n = 7), followed by Commune 4 (18.2%; n = 4). Communes 1, 2, and 8 each yielded three isolates (13.6% each), whereas Commune 6 yielded two isolates (9.1%). Two households were positive for *C. parapsilosis* in both monitoring periods. No isolates were recovered from Communes 5 and 7. The overall positivity rate was 15.4% at the household level (16/104) and 6.3% at the sample level (22/348).

### 3.2. Household Water Characteristics and Storage Practices

The majority of *C. parapsilosis* isolates (72.7%; n = 16) were recovered from household water storage tanks, with the remaining 27.3% (n = 6) originating from direct connections to the public water supply system. The pH of the positive water samples ranged from 4.0 to 7.0, with a mean of 5.4. Most positive samples had a pH between 5.0 and 6.0 (81.8%, n = 18). Overall, 90.5% of participating households had access to the public water supply system, and 81% stored water at home. Furthermore, 14 out of 21 households (66.7%) reported treating water before use, mainly by boiling or using commercial filtration systems.

### 3.3. Antifungal Susceptibility

The distribution of MIC values for amphotericin B, fluconazole, and anidulafungin against the 22 isolates of *C. parapsilosis* is presented in Table 1. Amphotericin B MIC values spanned four dilutions (0.03 µg/mL to 0.25 µg/mL), with MIC_50_ and MIC_90_ of 0.06 µg/mL and 0.12 µg/mL, respectively. The modal MIC was 0.06 µg/mL (n = 11). All isolates were inhibited at concentrations below the established CLSI ECV of 1 µg/mL.

Anidulafungin MIC values spanned five dilutions (0.015 µg/mL to 0.25 µg/mL), with MIC_50_ and MIC_90_ of 0.06 µg/mL and 0.25 µg/mL, respectively. The modal MIC was 0.015 µg/mL (n = 8), followed by 0.06 µg/mL (n = 6). All isolates were susceptible according to the CLSI clinical breakpoint of ≤2 µg/mL.

Fluconazole exhibited the widest MIC range among the three agents, spanning eight dilutions (0.06 µg/mL to 8 µg/mL), with MIC_50_ and MIC_90_ of 2 µg/mL and 4 µg/mL, respectively. The modal MIC was 4 µg/mL (n = 10). Notably, one isolate had an MIC of ≥8 µg/mL, which categorised it as resistant according to the CLSI clinical breakpoint. This resistant isolate was recovered from a household in Commune 3, originating from a water storage tank during the ascending rainfall period.

### 3.4. Detection of Mutations in ERG11

Among the 22 *C. parapsilosis* isolates recovered, one exhibited resistance to fluconazole. BLAST analysis of the *ERG*11 coding sequence from this isolate, aligned against the reference sequence (GenBank accession no. GQ302972), revealed a single non-synonymous substitution: an arginine-to-isoleucine change at codon 398 (R398I). No additional amino acid substitutions were detected in the *ERG*11 gene of this isolate.

## 4. Discussion

To the best of our knowledge, this is the first report describing the recovery of *C. parapsilosis* from intradomiciliary water in Colombia, and one of the few such reports for Latin America. The species was identified in 16 of 104 participating households (15.4%), with isolates recovered across both monitoring periods in equal numbers (11 isolates each) and no statistically significant difference between periods. This finding reinforces the notion that domestic water systems, rather than only clinical or hospital environments, may serve as reservoirs for this opportunistic pathogen [27].

The severe water supply disruptions experienced in Villavicencio during 2025 limited the availability of water directly from the distribution network and increased household reliance on water storage systems. Accordingly, the greater number of positive samples from storage tanks may partly reflect the larger number of samples collected from this source rather than a higher source-specific probability of contamination.

Nevertheless, household storage systems may provide conditions conducive to the persistence of *C. parapsilosis,* given the recognized ability of this species to adhere to and colonize plastic, rubber, and silicone surfaces, materials commonly found in household storage tanks and plumbing components [28,29,30]. Infrequently cleaned storage tanks may therefore constitute a persistent environmental niche that favors biofilm formation and prolonged survival of this yeast [31]. Indeed, *C. parapsilosis* has been described as a dominant contaminant of household appliances, and it is transferred via water to dishwashers, washing machines, and storage systems, where it subsequently proliferates [27].

The recovery of *C. parapsilosis* from domestic water in Colombia aligns with a growing body of evidence documenting the presence of this species in aquatic environments across the region. In Brazil, studies have reported *C. parapsilosis* from multiple water matrices: it was the predominant yeast species (68.5%) recovered from a hemodialysis unit water distribution system, and it was the most frequent species (43%) isolated from lagoon systems in Rio de Janeiro, being the only species recovered from all sampling points. Similarly, *C. parapsilosis* has been isolated from coastal waters in southern Brazil and from tropical freshwater environments in the southeastern region [32].

Perhaps the most clinically relevant finding of this study is the identification of a fluconazole-resistant *C. parapsilosis* isolate recovered from intradomiciliary water that carries a nonsynonymous R398I substitution in *ERG*11. Notably, this substitution has previously been reported in clinical *C. parapsilosis* isolates in combination with other mutations, such as Y132F and K143R, but has not been shown to independently confer azole resistance [33]. The mechanism underlying the resistance phenotype observed in this environmental isolate therefore remains undetermined. Resistance may be mediated by mechanisms not evaluated in the present study, such as *ERG*11 overexpression, efflux pump activity, or lipid-related adaptations, or the isolate may represent a borderline phenotype at the resistance breakpoint [34,35]. Comprehensive molecular and phenotypic characterization of this and additional environmental isolates is required to resolve this question. However, to our knowledge, this is the first report of azole-resistant *C. parapsilosis* recovered from a non-clinical domestic water source in Colombia.

This finding gains particular significance when contextualized with recent clinical data from Colombia. In a post-pandemic cohort study conducted at a high-complexity teaching hospital in Cali, 28.6% of *C. parapsilosis* bloodstream isolates were resistant to fluconazole [15]. Furthermore, a four-year surveillance study at a quaternary care hospital reported that 25% of *C. parapsilosis* clinical isolates displayed fluconazole resistance, with Erg11 amino acid substitutions (Y132F or K143R) identified in resistant strains [26]. The national laboratory-based surveillance led by the National Institute of Health in Colombia reveals up to 39% of fluconazole-resistant *C. parapsilosis* isolates [36].

Globally, fluconazole resistance in environmental *C. parapsilosis* has been documented with increasing frequency [14,37]. A recent study on potable water as a source of resistant strains reported that 5.8% of *Candida spp.* isolates showed borderline resistance to fluconazole, and *C. parapsilosis* from water samples had statistically higher MICs for anidulafungin than clinical strains [19]. Furthermore, environmental strains of *C. parapsilosis* have been found to harbor the same Erg11-Y132F amino acid substitution as clinical resistant isolates [33]. The presence of azole resistance in environmental isolates has been attributed to the widespread use of fluconazole in clinical settings and agriculture, as well as the discharge of antifungal residues into wastewater, which may exert selective pressure on aquatic yeast populations [38].

Our detection of a fluconazole-resistant *C. parapsilosis* isolate from a household water storage tank, combined with the high resistance rates reported in Colombian hospitals, raises concerns about the potential for environmental strains to serve as a reservoir for resistance or to directly contribute to human exposure through domestic water consumption.

Several limitations should be considered when interpreting these results. First, this investigation was nested within a broader water quality monitoring program primarily designed for bacterial pathogen detection; consequently, the culture media used (MacConkey agar and nutrient agar) were not optimized for fungal recovery, which may have underestimated the true prevalence and diversity of *Candida* species in the samples. Second, the study was limited to two monitoring periods (transition and ascending rainfall), which may not fully capture seasonal variations in yeast occurrence. Third, the sample size, while adequate for a descriptive cross-sectional study, was limited to 104 households in a single city, and the use of digital recruitment campaigns may have introduced selection bias. Finally, the absence of whole-genome sequencing or multilocus sequence typing prevents a definitive assessment of the genetic relatedness between environmental isolates and clinical strains circulating in Colombian hospitals.

Despite these limitations, this study provides the first evidence of *C. parapsilosis* in domestic water in Colombia and documents the presence of a fluconazole-resistant environmental isolate. These findings raise the possibility that domestic water systems may serve as environmental reservoirs for this opportunistic yeast; however, the clinical and public health implications remain to be determined.

## 5. Conclusions

This study provides the first evidence of *C. parapsilosis* in domestic water in Colombia and documents the presence of a fluconazole-resistant environmental isolate in a non-clinical setting. Although this finding is based on a single isolate recovered at the resistance breakpoint, it raises the possibility that azole-resistant *C. parapsilosis* may circulate outside hospital environments and that household water storage systems could serve as environmental reservoirs. These observations do not establish human exposure or transmission, and the clinical relevance of such environmental isolates remains undetermined. The results nevertheless support the need for further surveillance of domestic water supplies, for studies assessing water storage and treatment practices, and for molecular comparisons between environmental and clinical isolates to determine whether they are genetically related. Such investigations are required before the public health implications of these findings can be properly assessed.

## Figures and Tables

**Table 1:** Minimum inhibitory concentration (MIC) distributions and summary statistics for 22 environmental *Candida parapsilosis* isolates against three antifungal agents.

Species (n)	Antifungal	0.015	0.03	0.06	0.12	0.25	0.5	1	2	4	8	GM	MIC_50_	MIC_90_
*C. parapsilosis* (22)	amphotericin B	-	1	11	9	1	-	-	-	-	-	0.06	0.06	0.12
	anidulafungin	8	-	6	4	4	-	-	-	-	-	0.06	0.06	0.25
	fluconazole	-	-	4	1	-	-	1	5	10	1 *	1	2	4

* resistant isolate.

## Data Availability

The data supporting the findings of this study are available from the corresponding author upon reasonable request. The data are not publicly available because they contain sociodemographic and household-level information that could compromise participant privacy.
